# Aging as an essential modifier for the efficacy in mesenchymal stem cell therapy through an inositol phosphate 6 kinase-inositol pyrophosphate 7-dependent mechanism

**DOI:** 10.1186/scrt432

**Published:** 2014-03-26

**Authors:** Sherry Shuyi Wang, Jun Ren

**Affiliations:** 1 Center for Cardiovascular Research and Alternative Medicine, University of Wyoming College of Health Sciences, 1000 East University Avenue, Dept 3375, Laramie, WY 82071, USA

## Abstract

Mesenchymal stem cells (MSCs) are multipotent stromal cells originated from bone marrow and other adult tissues. MSCs are capable of differentiating into adipogenic, osteogenic, and chondrogenic lineages. Transplantation of bone marrow-derived MSCs has displayed some promise in the management against ischemic injuries such as myocardial infarction. Aging exhibited increased vulnerability of MSCs to hypoxic injury, higher inositol pyrophosphate 7 (IP7) levels, and decreased Akt phosphorylation. Inhibition of inositol hexakis phosphate kinases (IP6Ks) activates Akt signaling, decreases apoptosis, and modulates paracrine profiles in aged MSCs, and this greatly enhances the therapeutic efficacy of aged MSCs in the face of hypoxic injury.

## 

In this issue of *Stem Cell Research & Therapy*, Zhang and colleagues report that the advanced aging process drastically increases the vulnerability of mesenchymal stem cells (MSCs) to hypoxic injury [1]. These findings reveal a tie between pathological conditions such as aging and compromised MSC transplantation efficacy. Acute myocardial infarction (AMI) is the leading cause of morbidity and mortality in developed and developing countries. Rapid adoption of routine invasive strategies and intensive pharmacotherapy has been established to normalize coronary perfusion and enable viable ischemic tissue to recover from ischemic injury, thus reducing the mortality rate in patients with AMI [2,3]. Nonetheless, a significant portion of patients with AMI still develop left ventricular remodeling and heart failure with a subsequent high risk of mortality [4]. How to effectively restore heart function among patients with AMI remains a major clinical challenge. Ample clinical and experimental evidence during the past decades has depicted a role for stem cell therapy as a rather promising treatment avenue to facilitate myocardial function recovery after AMI. In particular, MSCs have been considered as a candidate for cardiac cell therapy because of their availability and plasticity [5]. Nonetheless, the poor survival and retention of implanted MSCs in the injury site because of the existence of a number of pathological conditions greatly limit the therapeutic potential of MSC therapy.

A previous study has demonstrated that the aging process may unfavorably affect the functional activity of stem cells and the tissue environment that surrounds them, thus limiting the therapeutic potential of MSCs [6]. Nonetheless, the mechanism behind decreased viability and impaired function of the aged engrafted MSCs remains unclarified. It was well perceived that the aging process directly affects cell-mediated improvement of neovascularization, revealing that young, but not old, bone marrow cells may be more readily incorporated into the neovasculature to restore cardiac angiogenic function [7]. Liang and colleagues [8] also noted a drastic decline in the therapeutic efficacy for old MSCs. In this issue of *Stem Cell Research & Therapy*, Zhang and colleagues report that aged MSCs exhibited higher apoptotic index, decreased Akt phosphorylation (Thr^308^), enhanced Bad activation, and decreased Bax/Bcl-2 ratio. Interestingly, inhibition of inositol hexakis phosphate kinases (IP6Ks) using the kinase inhibitor TNP overtly decreased inositol pyrophosphate 7 (IP7) production and relieved the MSC apoptotic index as evidenced by Akt phosphorylation (Thr^308^) and Bax/Bcl-2 ratio [1]. IP7, formed by a family of IP6Ks, represents a physiologic inhibitor of Akt which mediates survival signal. These authors examined the role of IP6K inhibition in the therapeutic efficacy of MSCs and its underlying mechanism. After introduction into an infarct heart, MSCs prevent deleterious remodeling and improve cardiac function, although a better understanding of MSC differentiation in the cardiac scar tissue is still at large [9]. The beneficial effect of MSCs is believed to be mediated in part through indirect paracrine actions, thus recruiting multiple therapeutic growth factors and cytokines to ischemic myocardium [10,11]. However, the overall efficacy of stem cell transplantation has been greatly hampered by several pathological conditions such as aging, diabetes, and obesity. One of the rather intriguing findings from Zhang and colleagues is the characterization of the paracrine profile of MSCs. Their results indicate that aging negatively modulates the paracrine profile of MSCs. In particular, overt reductions in the secretion of angiogenic factors are noted in aged MSCs, especially under hypoxia. These authors conclude that advanced aging may impair paracrine efficiency of MSCs at least in part by IP7 production. Inhibition of IP6K activity interrupts IP7 production and may represent a novel target for augmenting aged MSC therapeutic efficacy.

It is noteworthy that the overall efficacy of stem cell transplantation relies on the activity of donor cells and tissue environment. Novel approaches aiming at reversing dysfunction of transplanted cells or refreshing target tissues should provide a useful avenue for improvement of cell therapy efficacy in patients with AMI. The findings reported in this work [1] that inhibition of IP6Ks turns on Akt signaling, decreases apoptosis, and modulates the paracrine profile in MSCs should shed some light on a better strategy in promoting the therapeutic efficacy of MSCs in aging.

## Abbreviations

AMI: Acute myocardial infarction; IP6K: Inositol hexakis phosphate kinase; IP7: Inositol pyrophosphate 7; MSC: Mesenchymal stem cell; Thr308: Threonine 308.

## Competing interests

The authors declare that they have no competing interests.
